# Estimating Influenza Disease Burden from Population-Based Surveillance Data in the United States

**DOI:** 10.1371/journal.pone.0118369

**Published:** 2015-03-04

**Authors:** Carrie Reed, Sandra S. Chaves, Pam Daily Kirley, Ruth Emerson, Deborah Aragon, Emily B. Hancock, Lisa Butler, Joan Baumbach, Gary Hollick, Nancy M. Bennett, Matthew R. Laidler, Ann Thomas, Martin I. Meltzer, Lyn Finelli

**Affiliations:** 1 Influenza Division, Centers for Disease Control and Prevention, Atlanta, GA, United States of America; 2 California Emerging Infections Program, Oakland, CA, United States of America; 3 Colorado Emerging Infections Program, Colorado Department of Public Health and Environment, Denver, CO, United States of America; 4 New Mexico Emerging Infections Program, Santa Fe, NM, United States of America; 5 New York Emerging Infections Program, University of Rochester School of Medicine and Dentistry, Rochester, NY, United States of America; 6 Oregon Public Health Division, Portland, OR, United States of America; 7 Division Of Preparedness and Emerging Infections, Centers for Disease Control and Prevention, Atlanta, GA, United States of America

## Abstract

Annual estimates of the influenza disease burden provide information to evaluate programs and allocate resources. We used a multiplier method with routine population-based surveillance data on influenza hospitalization in the United States to correct for under-reporting and estimate the burden of influenza for seasons after the 2009 pandemic. Five sites of the Influenza Hospitalization Surveillance Network (FluSurv-NET) collected data on the frequency and sensitivity of influenza testing during two seasons to estimate under-detection. Population-based rates of influenza-associated hospitalization and Intensive Care Unit admission from 2010–2013 were extrapolated to the U.S. population from FluSurv-NET and corrected for under-detection. Influenza deaths were calculated using a ratio of deaths to hospitalizations. We estimated that influenza-related hospitalizations were under-detected during 2010-11 by a factor of 2.1 (95%CI 1.7–2.9) for age < 18 years, 3.1 (2.4–4.5) for ages 18-64 years, and 5.2 (95%CI 3.8–8.3) for age 65+. Results were similar in 2011-12. Extrapolated estimates for 3 seasons from 2010–2013 included: 114,192–624,435 hospitalizations, 18,491–95,390 ICU admissions, and 4,915–27,174 deaths per year; 54–70% of hospitalizations and 71–85% of deaths occurred among adults aged 65+. Influenza causes a substantial disease burden in the U.S. that varies by age and season. Periodic estimation of multipliers across multiple sites and age groups improves our understanding of influenza detection in sentinel surveillance systems. Adjusting surveillance data using a multiplier method is a relatively simple means to estimate the impact of influenza and the subsequent value of interventions to prevent influenza.

## Introduction

Influenza results in a significant clinical and economic impact each year [1]. Accurate estimates of the influenza disease burden provide public health officials with information to evaluate programs and allocate resources. Not every person who truly has influenza will seek medical care, be tested for influenza, have a positive test, and therefore be reported through influenza surveillance. Routinely available influenza diagnostic tests also vary in sensitivity. Thus, data collected through influenza surveillance and case finding represent only a fraction of persons infected with influenza.

The under-detection of influenza hospitalizations and deaths has traditionally been accounted for using statistical methods to model excess morbidity and mortality attributable to influenza using data from death certificates and medical encounters such as hospital discharge records [2–4]. These methods have been widely used over the past few decades in the United States (U.S.) and many other countries, but the data necessary to make estimates are often not available for 2–3 years following an influenza season.

To provide more timely influenza disease burden estimates during the spring wave of the 2009 H1N1 pandemic, the Centers for Disease Control and Prevention (CDC) developed a multiplier method to adjust case reports from state and local health departments in the U.S. for factors leading to under-detection of influenza [5]. This method was later expanded to use routine population-based influenza hospitalization surveillance data to make estimates of the number of influenza cases, hospitalizations, and deaths during the fall and winter pandemic wave as the pandemic unfolded [6].

Following the 2009 pandemic, there was continued interest by CDC in using existing population-based surveillance systems to provide estimates of the influenza disease burden during influenza seasons. Using multipliers estimated during the heightened awareness of a pandemic, however, may not accurately reflect non-pandemic seasons since influenza detection may differ during seasonal epidemics. To calculate multipliers that were more relevant to post-pandemic seasons, we collected data on the frequency and sensitivity of influenza testing during two seasons to correct for under-reporting in hospital surveillance. We used these data to estimate the disease burden attributable to influenza for three consecutive seasons following the 2009 pandemic.

## Materials and Methods

We based our method for estimating the U.S. influenza disease burden on annual surveillance data collected through the Influenza Hospitalization Surveillance Network (FluSurv-NET). This network conducts population-based sentinel surveillance for laboratory-confirmed influenza-associated hospitalizations from October to the following April in 13 geographically diverse surveillance areas across the U. S., covering approximately 9% of the country’s population. Surveillance officers identify all laboratory-confirmed influenza hospitalizations that occur among residents of the surveillance areas by monitoring 282 hospitals in 79 counties. Data from all sites are combined to report weekly during the influenza season on the level of influenza-associated hospitalizations by age group in the United States.

### Detection of influenza-associated hospitalizations

A patient is included in FluSurv-NET if he/she resides in the surveillance area and is admitted to a hospital in the catchment area with laboratory confirmation of influenza virus infection. Laboratory testing for influenza is ordered at the discretion of clinicians providing medical care, and confirmation may include a positive result from viral culture, direct or indirect fluorescent antibody (DFA/IFA), rapid antigen test (RAT), reverse transcription polymerase chain reaction (RT-PCR), or documentation of a positive test result in a patient’s medical record. Patients are identified through hospital laboratory and admission databases, infection control logs, and hospital discharge data for patients with a documented positive influenza test. Through medical record review, data are collected for each patient including demographic characteristics, medical history, and clinical course and outcomes (e.g., admission to intensive care unit, mechanical ventilation or death).

Because influenza testing in FluSurv-NET areas is performed at the discretion of the healthcare provider, a person hospitalized with influenza is only identified if s/he is tested for influenza and if the test correctly detects influenza. Patients with influenza are missed if they are not tested for influenza or if the tests used are not perfectly sensitive. To determine an appropriate multiplier to correct for under-detection of influenza hospitalizations, we collected additional data to estimate (a) the probability that a person who was hospitalized with a respiratory infection would have been tested for influenza, and (b) the probability that a person who truly had influenza would test positive for influenza (sensitivity of influenza testing).

To correct for under-detection of influenza hospitalizations we adjusted the reported rate of hospitalization for each age group by the proportion of patients tested for influenza and the average sensitivity of influenza testing. The overall level of under-detection was summarized using a multiplier that represents the expected number of true influenza hospitalizations per reported hospitalization and was calculated as:

$$Multiplier=\;\frac{1}{Frequency\;of\;influenza\;testing\;\times Sensitivity\;of\;influenza\;testing}$$

### Correction for under-detection of influenza-associated hospitalizations

Data collection was performed in a sample of participating surveillance areas to assess influenza testing practices among hospitalized patients with respiratory infections. Five surveillance areas (California, Colorado, New Mexico, New York, and Oregon) collected data during the periods December–April of the 2010–11 and 2011–12 seasons. The California surveillance area contributed data as two separate sites; California site 1 included facilities belonging to a large managed care organization that insures a large proportion of the surveillance population, while California site 2 included all other facilities in the surveillance area in northern California. Protocols were reviewed by human subjects specialists at CDC and local sites and determined to be public health surveillance and exempt from further IRB review. Patient information was de-identified prior to analysis.

To identify eligible persons hospitalized with a respiratory illness, sites selected hospitals that were representative of their catchment area and identified all patients who had been admitted with respiratory infection using a discharge audit of ICD-9 codes 466, 480–488. To determine the probability that a person hospitalized with a respiratory infection would have been tested for influenza, a stratified random sample of eligible patients per month by age group (<18 years, 18–64 years, and 65+ years) was selected to review laboratory records and/or medical charts and identify whether patients were tested for influenza and if so, what type of test was used. We analyzed data within age groups, but also examined whether there were other factors associated with influenza testing including month during the season, disease severity, or residual differences in finer age categories.

The sensitivity of influenza testing was calculated as a weighted average based on the distribution of test types performed at each site. A representative sensitivity value was selected for each test type from a review of the literature, with a lower estimate among persons aged 65+ based on studies that suggest lower sensitivity among older adults [7–9]: RAT (60% if age <65, 40% if age 65+), RT-PCR (90% if age <65, 85% if age 65+), Other (including culture or DFA/IFA); 70% if aged <65, 50% if age 65+). When multiple tests were performed for a single patient, the more sensitive test was used for all calculations.

To combine data across sites, simply pooling all observations into binomial estimates of each parameter has limitations. Numerous site-level factors contribute to variation in estimates from site to site, which can result in an incorrect summary estimate and its confidence interval. The beta-binomial method has been shown to appropriately combine heterogeneous studies to estimate summary frequencies [10]. The beta-binomial distribution is a binomial distribution in which the probability of success at each trial is not fixed but random and follows the beta distribution. For each season we applied beta-binomial models with maximum likelihood estimation to obtain pooled proportions using the SAS macro BETABIN [11]. This macro uses the SAS procedure NLMIXED to provide maximum likelihood estimates of the mean and standard deviation from each fitted distribution. Detailed explanation of the statistical assumptions for the beta-binomial model and the process of estimating the pooled proportions are described in Young-Xu et al. [10].

Independent models were fitted to estimate the pooled proportion of patients tested for influenza and the test sensitivity, and were hierarchical by age group. The estimated distributions for each parameter are shown in S1 Fig. The age-specific parameter estimates and their associated error were algebraically combined to calculate multipliers and 95% confidence intervals for the 2010–11 and 2011–12 seasons. We also calculated a summary multiplier for each age group following the same methods but using data from both seasons. Calculations were performed in SAS version 9.3 (Cary, NC).

### The ratio of deaths to hospitalizations

Data on the occurrence of death among hospitalized patients with influenza are captured in FluSurv-NET by medical record review and finalized at the end of the season. We used these data to calculate the risk of death among all influenza hospitalizations identified by age group and season.

Not all persons who die with influenza are admitted to a hospital prior to their death, and others may die after hospital discharge, thus hospital surveillance does not fully capture deaths due to influenza in the catchment area. To estimate a more complete ratio of deaths to hospitalizations, we also included data on the probability that a person with a respiratory infection would die outside of a hospital admission. For this we used publically available mortality data from the National Center for Health Statistics for the U.S. population in 2010 to identify the deaths attributable to pneumonia and influenza (ICD-10 codes: J10-J18) and the proportion that occurred while hospitalized vs. outside of a hospital admission (e.g., at home, on arrival, in the emergency department, in hospice or long-term care facility).

The ratio of deaths to hospitalizations (D:H) represents the expected number of influenza deaths relative to the number of influenza hospitalizations and was calculated algebraically for each age group as:

$$D:H=\;\frac{\#\;reported\;deaths}{\#\;reported\;hospitalizations}\times \frac{1}{\%\;of\;deaths\;that\;occur\;in\;hospital}$$

### Estimating the influenza disease burden

**Fig 1:**
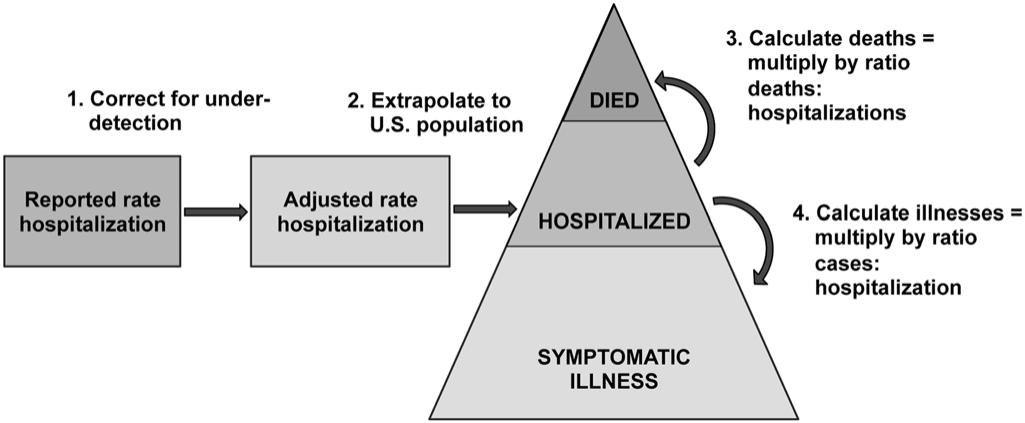
General framework for estimating influenza disease burden in the U.S. population using FluSurv-NET hospital-based influenza surveillance data.

Using an approach previously described in 2009 [6] and outlined in Fig. 1, we estimated the annual influenza disease burden by age group (<18 years, 18–64 years, 65+ years) using a series of age-specific parameters as described above: the rate of influenza hospitalization, the multiplier for under-detection (the probability of influenza testing and the sensitivity of influenza testing), the percent of influenza hospitalizations admitted to the ICU, and the ratio of deaths to hospitalizations. First, we adjusted the reported annual hospitalization rates from FluSurv-NET for three seasons from 2010–2013 using multipliers that included the probability of being tested for influenza and the sensitivity of influenza testing. Season-specific data were used for adjustments when available, and for the 2012–13 season summary data from the two measured seasons were used. Rates of influenza mortality were calculated by multiplying the adjusted rates of hospitalization by the ratio of deaths to hospitalizations. The rate of intensive care unit (ICU) admissions was calculated by multiplying the adjusted rate of hospitalization by the percent of hospitalized influenza patients admitted to the ICU per year in FluSurv-NET. The series of calculations were done as algebraic combinations of the observed values of each individual parameter and 95% confidence intervals were calculated by combining the associated uncertainties from all included parameters.

Lastly, adjusted rates of influenza-associated hospitalization, ICU admissions, and death and their 95% confidence intervals were applied to the annual U.S. population census estimates by age group to estimate the number of influenza-associated hospitalizations for each season. During the 2009 pandemic we also estimated the number of influenza illnesses in the population using a ratio of cases to hospitalizations based on data about medical care seeking and specimen collection, submission, and confirmation [5,6]. For the post-pandemic seasons, we lacked data on non-hospitalized illnesses and did not estimate this number of influenza illnesses in the population.

## Results

From December 2010 through April 2011, sites reported data from medical records on influenza testing practices for 5,458 patients hospitalized with a respiratory infection (ICD-9 codes: 466, 480–488). Two sites’ data included all patients (California site 1, Oregon); other sites included an age-stratified random sample of 60 charts per month. From December 2011 through April 2012, sites reported data from chart review on influenza testing practices for 2,506 patients. One site included data for all patients (California site 1); other sites included an age-stratified random sample of 60 charts per month.

**Fig 2:**
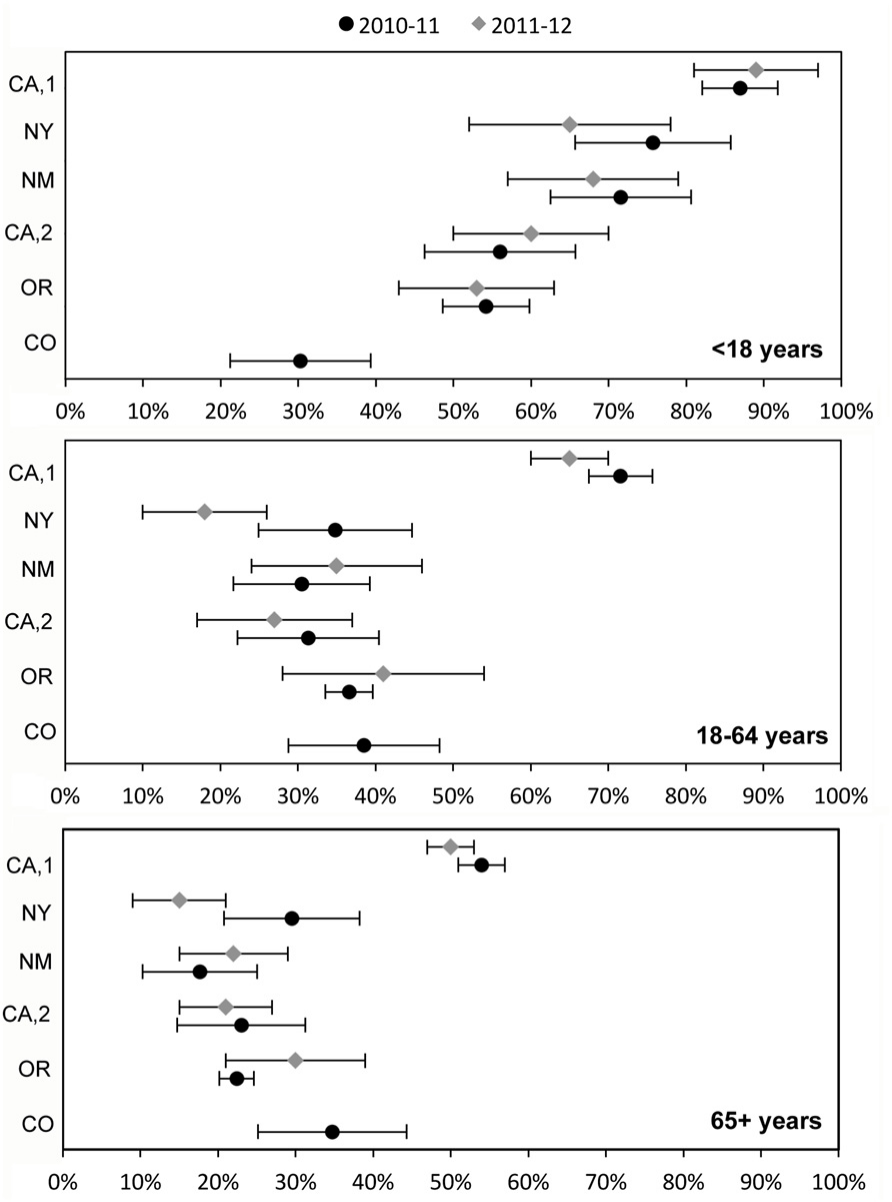
Proportion of patients tested for influenza (with binomial 95% confidence interval) by age group among hospitalized patients with a respiratory infection* in six FluSurv-NET sites.

The proportion of patients tested for influenza varied considerably by site and season but generally decreased with age: 30–89% among children aged <18 years, 18–72% among younger adults aged 18–64 years, and lowest at all sites among older adults aged 65+ years ranging 15–50% (Fig. 2, S1). Testing did not further vary within age groups except among the younger adults, with testing being less common among adults closer to age 65 years. There was some variation in influenza testing by month (S2 Fig.). The median percent of patients tested for influenza across sites increased from 30% in December to 48% in March during 2010–11, and from 32% in December to 42% in April during 2011–12, with greater variability among adults than children. In a logistic regression model controlling for age and site, patients who died were 0.71 (95% CI: 0.60–0.84) times as likely to have been tested for influenza as patients who survived to discharge.

**Fig 3:**
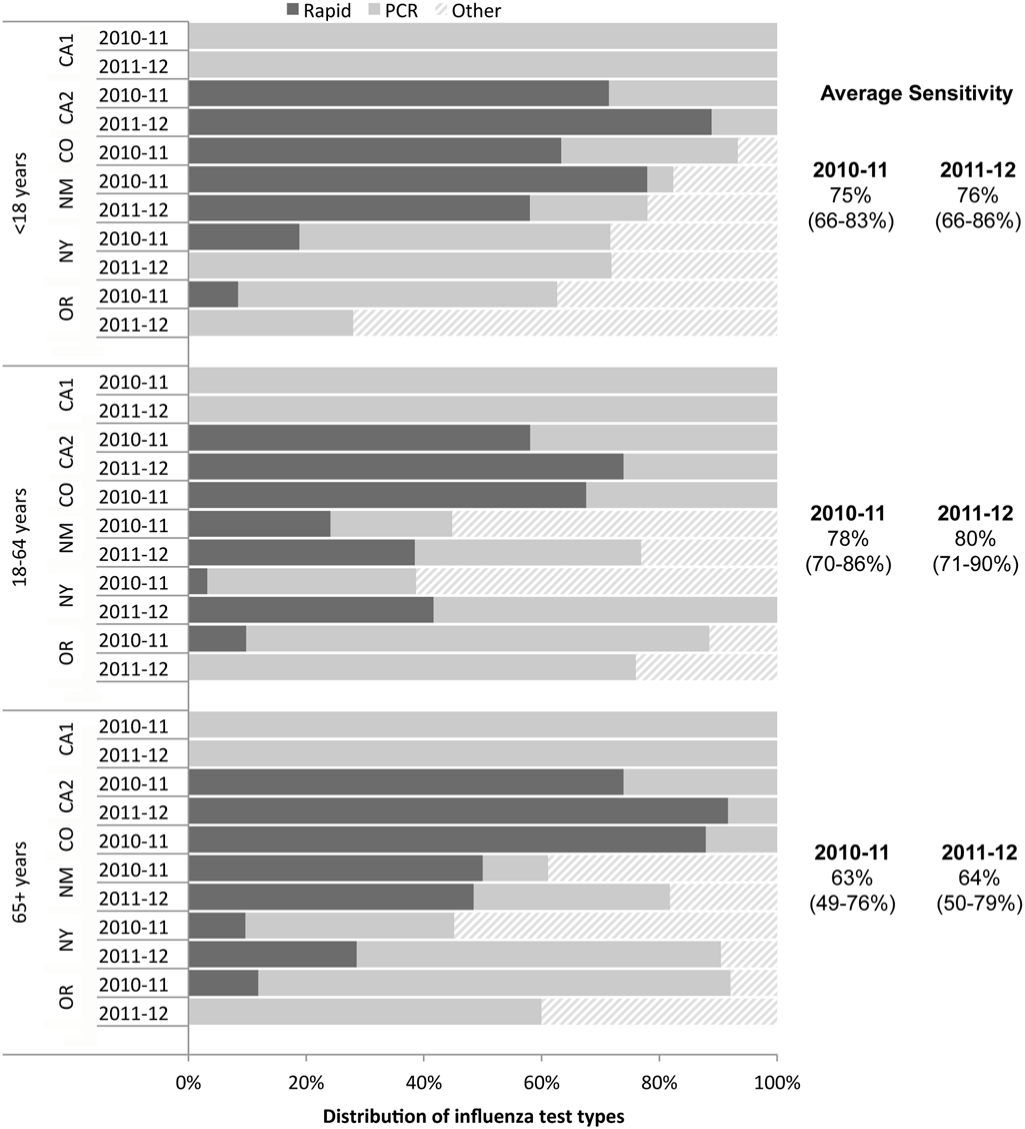
Distribution of influenza test types used and average sensitivity of influenza testing among hospitalized patients tested for influenza in six FluSurv-NET sites.

The sensitivity of influenza testing also varied between sites, depending on the mix of test types performed. Rapid tests and RT-PCR were the most commonly performed tests overall, though the distribution of test types varied substantially from site to site (Fig. 3). There was less variation by age, though in several sites children were more likely to be tested with rapid tests than adults. There are likely facility-level differences within a surveillance area; for example, the California site belonging to a major managed care plan used exclusively RT-PCR testing, while the other facilities in that area used predominantly rapid tests.

We combined the frequency and sensitivity of influenza testing from six sites to estimate a multiplier for FluSurv-NET under-detection of 2.0–5.6, varying by season and age group, with the highest magnitude of correction needed among adults aged 65+ years (Table 1). Multipliers for individual sites ranged from a low of 1.2 among children in one site to a high of 10.8 among older adults in another. For a given age group, there was less variability between seasons, and the summary multiplier for each age group was similar when the two seasons were combined.

**Table 1:** Estimated multiplier for under-detection of influenza among hospitalized persons in FluSurv-NET.

		<18 years	18–64 years	65+ years
**2010–11**	Estimated multiplier (95%CI)	2.1 (1.7–2.9)	3.1 (2.4–4.5)	5.2 (3.8–8.3)
	Multiplier by site, range	1.3–4.7	1.6–5.0	2.2–11.6
	Multiplier by month, range	1.9–2.4	2.4–4.7	3.3–7.8
**2011–12**	Estimated multiplier (95%CI)	2.0 (1.6–2.6)	3.3 (2.4–5.4)	5.6 (3.9–9.8)
	Multiplier by site, range	1.2–3.1	1.7–7.9	2.4–10.8
	Multiplier by month, range	1.8–2.0	2.8–3.6	3.9–6.6
Summary*	Estimated multiplier (95%CI)	2.1 (1.8–2.6)	3.2 (2.6–4.3)	5.3 (4.1–7.8)

The multiplier represents the number of estimated true influenza hospitalizations per reported hospitalization.

*Summary multipliers were calculated using the same methods as 2010–11 and 2011–12 but using all the data from both seasons.

Across all FluSurv-NET sites, the risk of ICU admission among hospitalized patients with influenza was relatively consistent between years and age groups, varying from 15–19% (Table 2). The risk of death during influenza hospitalizations increased with age, from 0.2–0.9% of children <18 years to 1.8–2.8% of younger adults 18–64 years, and 3.4–4.7% of older adults 65+ years. Deaths among children were least likely to be captured by hospital-based surveillance, with ~50% of death certificates for children with pneumonia & influenza indicating that death occurred outside of a hospital admission, compared with ~25% of younger adults and ~30% of older adults.

**Table 2:** Risk of severe influenza-associated outcomes among persons hospitalized with influenza by season and age group.

	Age group	Risk ICU admission, if hospitalized*	Risk of death, if hospitalized*	% deaths in hospital**	Ratio D:H (95% CI)***
	<18 years	15.7%	0.3%	49.5%	0.006 (0.001–0.012)
**2010–11**	18–64 years	18.9%	2.8%	74.2%	0.037 (0.029–0.046)
	65+ years	16.1%	4.7%	68.7%	0.068 (0.055–0.081)
	<18 years	19.4%	0.2%	43.6%	0.005 (0.002–0.009)
**2011–12**	18–64 years	16.1%	2.8%	74.7%	0.038 (0.023–0.052)
	65+ years	15.9%	3.4%	67.5%	0.050 (0.033–0.068)
	<18 years	18.2%	0.9%	46.2%	0.020 (0.010–0.030)
**2012–13**	18–64 years	16.6%	1.8%	74.5%	0.024 (0.018–0.029)
	65+ years	14.7%	3.6%	68.1%	0.053 (0.046–0.060)

* Calculated from FluSurv-NET surveillance data, all sites

**Among deaths coded as pneumonia, influenza, or acute lower respiratory tract infections in the 2010 and 2011 National Multiple Cause Mortality Files. 2012–13 is an average of the two years with available data.

*** Algebraic combination of the risk of death if hospitalized and the % of deaths that occur during a hospital admission. 95% confidence intervals were calculated by combining the associated binary uncertainty of each parameter.

Applying our results to the U.S. population by age group for each of the three post-pandemic seasons, we estimated 114,018–633,001 total hospitalizations, 18,476–96,667 ICU admissions, and 4,866–27,810 deaths per season (Table 3). The three included seasons varied in the timing and intensity of influenza activity (S2 Fig.) with the highest estimates seen for the 2012–13 season and the lowest for the 2011–12 season. Because of the variability in influenza testing during the season, we repeated our estimates with multipliers stratified by month (Table 1) and found little difference in the estimated numbers of hospitalizations, ICU admissions, or deaths per season.

**Table 3:** Estimates of influenza disease burden in rates per 100,000 and number per U.S. population by age group and influenza season, United States.

		Rate (95% confidence interval)			Number (95% confidence interval)		
		2010–11	2011–12	2012–13	2010–11	2011–12	2012–13
**Hospitalizations**	<18 yrs	**41.8**	**13.5**	**56.8**	**30,954**	**10,005**	**41,887**
		(31.0–52.6)	(10.1–17.0)	(45.8–67.9)	(22,948–38,960)	(7,433–12,578)	(33,736–50,038)
	18–64 yrs	**43.7**	**17.1**	**73.8**	**85,183**	**33,589**	**145,331**
		(30.4–57.1)	(10.3–23.9)	(55.3–92.2)	(59,249–111,117)	(20,241–46,938)	(109,040–181,622)
	65+ yrs	**335**	**170**	**1,033**	**135,626**	**70,423**	**445,783**
		(208–462)	(96–245)	(712–1,355)	(84,297–186,954)	(39,606–101,240)	(307,023–584,544)
	Total	**81.4**	**36.6**	**202**	**251,763**	**114,018**	**633,001**
		(53.8–109)	(21.6–51.6)	(143–260)	(166,494–337,031)	(97,280–160,756)	(449,789–816,204)
**ICU**	<18 yrs	**6.5**	**2.6**	**10.3**	**4,854**	**1,934**	**7,568**
		(4.7–8.4)	(1.8–3.5)	(8.0–12.5)	(3,460–6,249)	(1,316–2,552)	(5,921–9,215)
	18–64 yrs	**8.2**	**2.7**	**12.2**	**16,038**	**5,392**	**24,001**
		(5.6–10.8)	(1.6–3.9)	(9.0–15.3)	(10,988–21,089)	(3,106–7,678)	(17,788–30,214)
	65+ yrs	**53.9**	**27.0**	**151**	**21,826**	**11,151**	**65,098**
		(32.8–75.0)	(14.5–39.4)	(103–199)	(13,286–30,365)	(6,002–16,300)	(44,449–85,747)
	Total	**13.8**	**5.9**	**30.8**	**42,718**	**18,476**	**96,667**
		(9.0–18.7)	(3.3–8.5)	(21.7–39.9)	(27,733–57,703)	(10,424–26,529)	(68,157–125,176)
**Deaths**	<18 yrs	**0.26**	**0.07**	**1.1**	**190**	**52**	**841**
		(0.01–0.50)	(0.02–0.12)	(0.55–1.7)	(10–369)	(13–90)	(404–1,279)
	18–64 yrs	**1.6**	**0.6**	**1.7**	**3,183**	**1,267**	**3,418**
		(1.0–2.3)	(0.29–1.0)	(1.2–2.3)	(1,969–4,398)	(571–1,963)	(2,263–4,574)
	65+ yrs	**22.8**	**8.6**	**54.6**	**9,232**	**3,547**	**23,551**
		(13.1–32.5)	(3.8–13.3)	(36.2–73.0)	(5,295–13,169)	(1,580–5,514)	(15,599–31,502)
	Total	**4.1**	**1.6**	**8.9**	**12,605**	**4,866**	**27,810**
		(2.4–5.8)	(0.7–2.4)	(5.9–12.1)	(7,275–17,935)	(2,164–7,567)	(18,266–37,354)

Older adults aged 65+ years accounted for 54–70% of hospitalizations and 73–85% of deaths depending on the season, and had the highest rates of hospitalization (170–1,033 per 100,000 persons) and death (8.6–55 per 100,000). By comparison, children had lower rates of hospitalization (14–57 per 100,000 persons) and especially death (0.26–1.1 per 100,000), followed by younger adults (hospitalization: 17–74 per 100,000 persons; death: 0.6–1.7 per 100,000 persons).

## Discussion

From the 2010–11 through 2012–13 seasons, we estimated that approximately 115,000–630,000 influenza-associated hospitalizations, 18,000–96,000 ICU admissions, and 5,000–27,000 deaths occurred in the U.S., depending on the season. These estimates are similar to previously published national estimates of 86,494–544,909 hospitalizations per year from the years 1979–2001[4] and 3,349–48,614 deaths per year from 1976–2007 [2] using models of excess influenza-associated morbidity and mortality. After accounting for the under-detection of influenza, our estimates represented 2.0–5.6 times the level of influenza morbidity as reported by influenza hospitalization surveillance. Under-detection of influenza varied substantially with the patient’s age, highest among older adults, who were least likely to be tested for influenza during hospitalization. The degree of under-detection also varied by site, up to ten times the level of reported hospitalization in some sites for older adults, but was relatively stable between the 2010–11 and 2011–12 influenza seasons.

There are practical limitations to influenza surveillance that often lead to detected cases being the “tip of the iceberg”. For comparison, we previously estimated a multiplier during the 2009 pandemic of 2.7 times (range: 1.7–4.5) the number of influenza hospitalizations reported to CDC, though we did not have sufficient data to stratify by age group [5]. Previous studies found a similar degree of under-detection among children during two seasons prior to the 2009 pandemic, estimating that clinical laboratory-based influenza detection captured 38% and 39% of pediatric influenza hospitalizations in one surveillance area [12,13]. These estimates are both similar to our post-pandemic estimate among children, though data are lacking on comparable estimates of the sensitivity of influenza surveillance among adults. While the under-recognized burden of influenza has been better documented among children [14], we found that adults, especially older adults, with influenza were even less likely to be identified through surveillance.

Our method provides a straightforward approach to better characterize influenza disease burden that could be done in a timely manner with routine surveillance data using only a few additional pieces of data that can be measured during influenza seasons. In recent years, various methods have been used to try to correct surveillance data for under-detection during influenza seasons [15], pandemics [16], and novel influenza events such as H3N2v in the United States [17], and H7N9 in China [18]. These methods vary from simple multipliers to more complex mathematical and statistical models depending on the context and available data. Where possible, it will be important to compare and evaluate these methods in light of their strengths and limitations. All of these methods, however, highlight the need to understand the processes that determine how persons are identified by surveillance systems in order to appropriately adjust for the biases that may be present.

The three included seasons varied substantially in timing and intensity, as well as the distribution of influenza types and subtypes [19–21], with an over five-fold difference in estimated influenza disease burden between a relatively mild season in 2011–12 and the more severe 2012–13 season. The substantial annual variation in influenza disease burden we estimated over three seasons was consistent with all other surveillance indicators in the United States and emphasizes the importance of having timely disease burden estimates during or shortly following an influenza season to provide public health officials with information to evaluate annual programs and allocate resources. Notably, our annual estimates of influenza disease burden are used to estimate and communicate the population impact of the influenza vaccination program in the U.S. [22,23]. The estimates previously relied on the multipliers calculated during the pandemic, which were assumed to be constant across age groups and lower than we found here, and therefore likely underestimated the influenza disease burden and number of outcomes averted by vaccination.

Our analysis was subject to some limitations. First, we assumed that the probability of a person with influenza being tested for influenza was the same as all persons with a respiratory illness. If physicians were more likely to recognize influenza patients clinically and select those patients for testing, we may have over-estimated the magnitude of under-detection. Second, to reduce complexity we estimated the average sensitivity of influenza testing using fixed values of test sensitivity that were informed by the literature. There is variation across reports of test sensitivity and sparse age-specific data, thus our estimates may not fully reflect the level of uncertainty due to diagnostic test sensitivity. However, preliminary analysis indicated that the large variation between sites in the proportion of rapid tests vs. RT-PCR seemed to have a larger influence on the overall uncertainty than the variability in individual test sensitivity.

Third, testing practices were assessed among persons who had ICD-9 codes that indicated pneumonia or influenza as over 90% of patients identified in the surveillance system had an ICD-9 discharge code of pneumonia or influenza. Persons with influenza can also have other respiratory or circulatory complications [24] though only 3% of patients in this surveillance system had no respiratory ICD-9 code. Our estimates of influenza-associated hospitalizations and deaths thus represent those with a respiratory presentation and may underestimate the full burden of severe influenza, especially among older adults, who may present with cardiovascular and other circulatory complications. Likewise, our estimate of deaths may also be underestimated because we did not adjust for the finding that patients who died in the hospital were less likely to have been tested for influenza than other hospitalized patients. Other methods, such as statistical models of excess influenza mortality [3], will continue to also be used to estimate influenza-related deaths in the U.S. for comparison, but this method allows for an interim estimate of respiratory influenza deaths that may be available earlier in a season than other methods that rely on time series modeling. Finally, we applied the rates from FluSurv-NET to the whole U.S. population to estimate national numbers of influenza-associated outcomes per year. While the 13 states are geographically diverse, they cover ~9% of the U.S. population and tend to be in more populous areas of their respective states and thus could have influenza rates that differ from the rest of the U.S. population.

Population-based sentinel surveillance is a valuable tool for monitoring the annual disease burden attributable to influenza around the world, but can be an underestimate. Data collected in two post-pandemic seasons for multiple sites and age groups allow us to better characterize influenza detection and reporting in the U.S. Periodic evaluation of this method is needed to examine how detection probabilities may vary in future seasons and how often multipliers should be recalculated. The multiplier method allows for a relatively simple means of correcting and extrapolating surveillance data to estimate the annual influenza disease burden and could be adapted to other countries that have population-based surveillance for severe influenza. Having timely estimates of the influenza disease burden in a population is important for public health decision-making, and will continue to be needed in the U.S. to evaluate and communicate the impact of influenza disease and possibilities for intervention, such as influenza vaccination, in the population.

## Supporting Information

S1 Fig Beta-binomial probability distributions of the summary proportion of patients tested for influenza and sensitivity of influenza testing across six FluSurv-NET sites, by age group and year. (TIF)

S2 Fig Proportion of patients tested for influenza by month and season among persons hospitalized with respiratory infection across six FluSurv-NET sites. (TIF)

S1 Table Proportion of patients tested for influenza (with 95% confidence interval) among hospitalized patients with a respiratory infection* in participating sites. (DOCX)

S2 Table Distribution of influenza test types used and average sensitivity of influenza testing among hospitalized patients tested for influenza in five participating sites. (DOCX)
