# Supplementary figures and images for: Estimating Influenza Disease Burden from Population-Based Surveillance Data in the United States
Source: PLoS One. 2015 Mar 4;10(3):e0118369. doi: 10.1371/journal.pone.0118369 (PMC4349859; doi:10.1371/journal.pone.0118369)

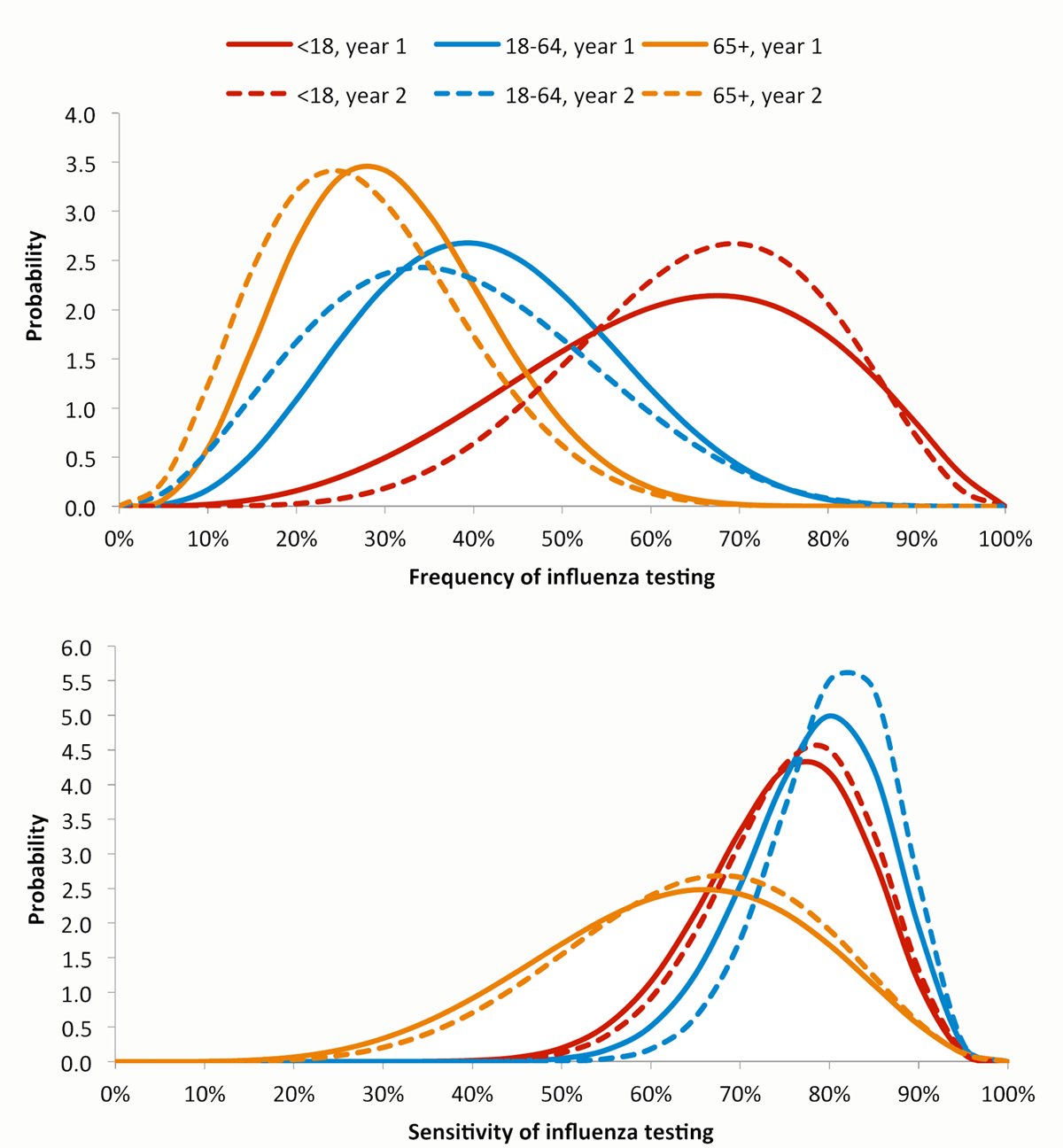

Supplement: S1 Fig — (TIF) [file pone.0118369.s001.tif]

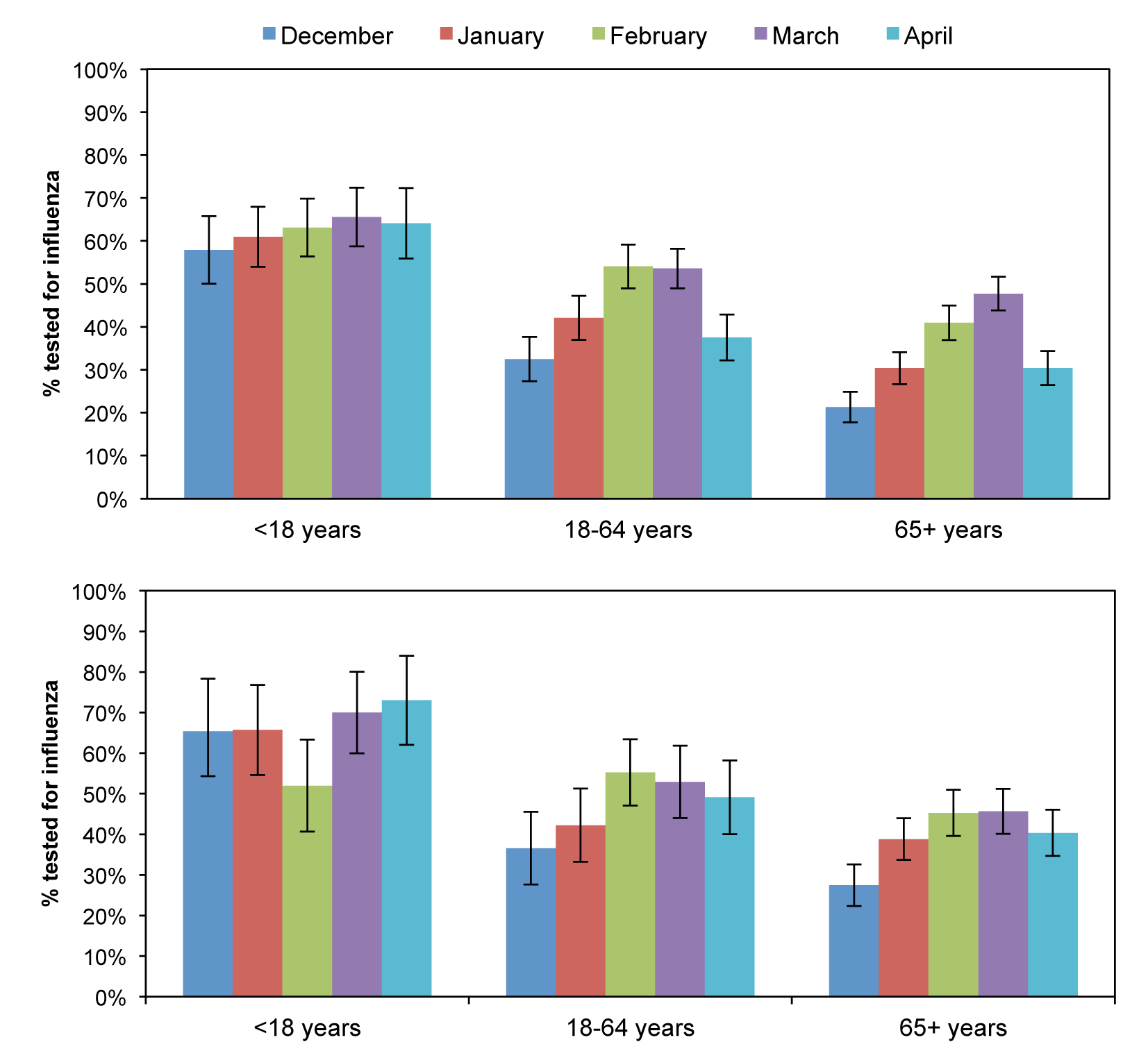

Supplement: S2 Fig — (TIF) [file pone.0118369.s002.tif]
