# Supplementary material for: Estimating Influenza Disease Burden from Population-Based Surveillance Data in the United States
Source: PLoS One. 2015 Mar 4;10(3):e0118369. doi: 10.1371/journal.pone.0118369 (PMC4349859; doi:10.1371/journal.pone.0118369)
Supplement: S1 Table — (DOCX) [file pone.0118369.s003.docx]

**Table S1. Frequency of influenza testing (with 95% confidence interval) among hospitalized patients with a respiratory infection* in participating sites.**

|  |  | **2010-11** | |  | **2011-12** | |
| --- | --- | --- | --- | --- | --- | --- |
| **Age group / Site** | **n** | **% tested** | **(95% CI)** | **n** | **% tested** | **(95% CI)** |
| **<18 years** |  |  |  |  |  |  |
| California, 1 | 184 | 87% | (82–92%) | 65 | 89% | (81–97%) |
| California, 2 | 100 | 56% | (46–66%) | 90 | 60% | (50–70%) |
| Colorado | 99 | 30% | (21–39%) | - | - |  |
| New Mexico | 95 | 72% | (63–81%) | 75 | 68% | (57–79%) |
| New York | 70 | 76% | (66–86%) | 49 | 65% | (52–79%) |
| Oregon | 306 | 54% | (49–60%) | 61 | 41% | (28–54%) |
| *Combined* |  | 62% | (48–77%) |  | 66% | (52–79%) |
| **18-64 years** |  |  |  |  |  |  |
| California, 1 | 461 | 72% | (67–76%) | 313 | 65% | (60–70%) |
| California, 2 | 99 | 31% | (22–41%) | 86 | 27% | (17–36%) |
| Colorado | 96 | 39% | (29–48%) | - | - |  |
| New Mexico | 105 | 28% | (19–36%) | 74 | 35% | (24–46%) |
| New York | 89 | 35% | (25–45%) | 74 | 18% | (10–27%) |
| Oregon | 951 | 37% | (34–40%) | 61 | 41% | (28–54%) |
| *Combined* |  | 41% | (30–53%) |  | 38% | (23–52%) |
| **65+ years** |  |  |  |  |  |  |
| California, 1 | 1,049 | 54% | (51–57%) | 961 | 50% | (47–53%) |
| California, 2 | 100 | 23% | (15–31%) | 175 | 21% | (15–27%) |
| Colorado | 95 | 35% | (25–44%) | - | - |  |
| New Mexico | 102 | 18% | (10–25%) | 151 | 22% | (15–28%) |
| New York | 105 | 30% | (21–38%) | 149 | 15% | (9–21%) |
| Oregon | 1,352 | 22% | (20–25%) | 118 | 30% | (21–38%) |
| *Combined* |  | 31% | (21–40%) |  | 28% | (23–52%) |

*ICD-9 discharge codes included: 466, 480-488
