# Supplementary material for: Estimating Influenza Disease Burden from Population-Based Surveillance Data in the United States
Source: PLoS One. 2015 Mar 4;10(3):e0118369. doi: 10.1371/journal.pone.0118369 (PMC4349859; doi:10.1371/journal.pone.0118369)
Supplement: S2 Table — (DOCX) [file pone.0118369.s004.docx]

**Table S2. Distribution of influenza test types used and average sensitivity of influenza testing among hospitalized patients tested for influenza in five participating sites.**

| **Age group / Site** | **% Rapid** | | **% RT-PCR** | | **% Other** | | **2010-11 sensitivity*** | **2011-12 sensitivity*** |
| --- | --- | --- | --- | --- | --- | --- | --- | --- |
| **<18 years** | **2010-11** | **2011-12** | **2010-11** | **2011-12** | **2010-11** | **2011-12** |  |  |
| California, 1 | 0 | 0 | 100 | 100 | 0 | 0 | 90% | 90% |
| California, 2 | 71 | 85 | 29 | 15 | 0 | 0 | 69% | 63% |
| Colorado | 63 | - | 30 | - | 7 | - | 70% | - |
| New Mexico | 78 | 58 | 4 | 20 | 18 | 22 | 63% | 68% |
| New York | 19 | 0 | 53 | 72 | 28 | 28 | 79% | 84% |
| Oregon | 8 | 0 | 54 | 28 | 37 | 72 | 80% | 75% |
| *Combined (95%CI)* | | | | | | | *75% (66–83%)* | *76% (66–86%)* |
| **18-64 years** | **2010-11** | **2011-12** | **2010-11** | **2011-12** | **2010-11** | **2011-12** |  |  |
| California, 1 | 0 | 0 | 100 | 100 | 0 | 0 | 90% | 90% |
| California, 2 | 58 | 71 | 42 | 29 | 0 | 0 | 73% | 67% |
| Colorado | 68 | - | 32 | - | 0 | - | 70% | - |
| New Mexico | 24 | 38 | 21 | 38 | 55 | 23 | 72% | 73% |
| New York | 3 | 42 | 35 | 58 | 61 | 0 | 77% | 77% |
| Oregon | 10 | 0 | 79 | 76 | 12 | 24 | 85% | 85% |
| *Combined (95%CI)* | | | | | | | *78% (70–86%)* | *80% (71–90%)* |
| **65+ years** | **2010-11** | **2011-12** | **2010-11** | **2011-12** | **2010-11** | **2011-12** |  |  |
| California, 1 | 0 | 0 | 100 | 100 | 0 | 0 | 52% | 85% |
| California, 2 | 74 | 92 | 26 | 8 | 0 | 0 | 77% | 43% |
| Colorado | 88 | - | 12 | - | 0 | - | 45% | - |
| New Mexico | 50 | 45 | 11 | 33 | 39 | 18 | 49% | 56% |
| New York | 10 | 28 | 35 | 62 | 55 | 10 | 61% | 68% |
| Oregon | 12 | 0 | 80 | 60 | 8 | 40 | 77% | 71% |
| *Combined (95%CI)* | | | | | | | *63% (49–76%)* | *64% (50–79%)* |

* Weighted average, based on site distribution of test types. Assumed sensitivity of test types: Rapid antigen test (RAT) (60% if age <65, 40% if age 65+), reverse transcription polymerase chain reaction (RT-PCR) (90% if age <65, 85% if age 65+), Other (including culture or direct/indirect fluorescent antibody (DFA/IFA); 70% if aged <65, 50% if age 65+)
